# Persistence Increases in the Absence of the Alarmone Guanosine Tetraphosphate by Reducing Cell Growth

**DOI:** 10.1038/srep20519

**Published:** 2016-02-03

**Authors:** Nityananda Chowdhury, Brian W. Kwan, Thomas K. Wood

**Affiliations:** 1 Department of Chemical Engineering, Pennsylvania, Pennsylvania State University, University Park, 16802-4400, USA; 2 Department of Biochemistry and Molecular Biology, Pennsylvania State University, University Park, Pennsylvania, 16802-4400, USA

## Abstract

Most bacterial cells are stressed, and as a result, some become tolerant to antibiotics by entering a dormant state known as persistence. The key intracellular metabolite that has been linked to this persister state is guanosine tetraphosphate (ppGpp), the alarmone that was first linked to nutrient stress. In *Escherichia coli,* ppGpp redirects protein production during nutrient stress by interacting with RNA polymerase directly and by inhibiting several proteins. Consistently, increased levels of ppGpp lead to increased persistence; but, the mechanism by which elevated ppGpp translates into persistence has not been determined. Hence, we explored persistence in the absence of ppGpp so that the underlying mechanism of persister cell formation could be explored. We found that persister cells still form, although at lower levels, in the absence of ppGpp. Additionally, the toxin/antitoxin systems that we investigated (MqsR, MazF, GhoT, and YafQ) remain able to increase persistence dramatically in the absence of ppGpp. By overproducing each *E. coli* protein from the 4287 plasmid vectors of the ASKA library and selecting for increased persistence in the absence of ppGpp (via a *relA spoT* mutant), we identified five new proteins, YihS, PntA, YqjE, FocA, and Zur, that increase persistence simply by reducing cell growth.

Microbial infections are the leading cause of death worldwide^1^, and one of the main causes of these recurring infections are persister cells^2^, those cells that are highly tolerant to traditional antibiotics. This tolerance to antibiotics is not due to genetic change^3^ but instead due to metabolic inactivity as demonstrated by both the initial discoverers of persisters^3^,^4^ and by a subsequent study showing a reduction in protein translation and ATP production converts nearly all exponentially-growing cells to persister cells^5^. Persister cells may arise stochastically^6^ but are formed primarily through environmental influence^5^,^7^,^8^,^9^,^10^,^11^.

The mechanism of persister cell formation has been elusive in that deletion studies have yielded little insight to date^12^, but two common elements are dependence on guanosine tetraphosphate (ppGpp) and on toxins of toxin/antitoxin (TA) systems. In *Escherichia coli,* ppGpp is produced as a response to nutrient starvation and other stresses (e.g., acid stress) and serves to shift transcription away from loci required for rapid growth and toward those needed for stress survival^13^. In *E. coli*, ppGpp is produced via RelA and SpoT (which can also degrade ppGpp)^13^,^14^. RelA and SpoT homologs involved in ppGpp metabolism have been characterized in other bacteria as well^14^. ppGpp alters transcription via interactions with RNA polymerase and by activation of RpoS (sigma^S^), the stress response sigma factor for the stationary phase, and RpoE (sigma^E^), the stress response sigma factor for misfolded proteins in the periplasm^13^. ppGpp also directly reduces DNA replication and protein synthesis^13^,^14^. Generally, the function of ppGpp as a repressor or activator of target genes is dictated by the presence of GC-rich or AT-rich sequences present in the promoter region^14^.

TA systems are found in most bacteria and are primarily used by the cell to reduce metabolism^15^. TA systems were first linked to persistence in 1983^16^ through mutagenesis and the identification of high persistence (*hip*) mutant *hipA7* that increases persistence. The *hipBA* locus encodes a toxin/antitoxin module, in which the HipA toxin phosphorylates the active center of the aminoacyl-tRNA synthetase GltX to inhibit translation^17^; however, the two substitutions of the HipA7 toxin variant (G22S and D291A) render the protein non-toxic, so the mechanism by which HipA7 increases persistence is not known and is not via increased toxicity of a TA pair^18^. Other links between TA systems and persistence include that production of most toxins increase persistence; for example, production of toxin RelE increases persistence 10,000-fold^19^. Also, deleting several individual TA loci has been shown to reduce persistence: *mqsR* and *mqsRA*^20^,^21^,^22^, *tisAB/istR*^7^, and *yafQ*^23^. Critically, ppGpp was linked to toxin/antitoxins in 1996^24^ since ppGpp is required for MazF toxicity (MazF is an endonuclease toxin of the type 2 MazF/MazE TA system), and ppGpp was shown in 2003^18^ to be required for persistence of the HipA7 variant. Therefore, ppGpp and TA systems are intimately linked to persistence.

The mechanism by which ppGpp activates toxins of TA systems has not been determined definitively. It has been proposed that production of polyphosphate, due to elevated ppGpp levels resulting from stochastic events, activates Lon protease and that activated Lon then degrades antitoxins, which would result in activated toxins^25^. However, *in vitro*, Lon is not activated by polyphosphate but instead is deactivated by it^26^, and deletions in *lon* have no effect on persistence against aminoglycosides or β-lactams^12^. Furthermore, deleting *ppk*, which encodes the enzyme that produces polyphosphate, has little impact on persistence against aminoglycosides^12^. Oddly, for the YoeB/YefM TA system, one of the TA systems upon which the polyphosphate model is based^25^, degradation of YefM antitoxin has been shown by the same lab in a subsequent publication to be independent of ppGpp and polyphosphate^27^. Critically, this model^25^ also neglects persister formation in response to environmental stress since it relies solely on stochastic generation of persister cells^28^. Additionally, although ppGpp is required, Lon protease and polyphosphate are not related to persistence that stems from activation of TA systems in which the antitoxin is an antisense RNA rather than a protein^28^. Therefore, the link between polyphosphate and toxin activation via Lon protease is controversial, and the underlying mechanisms for persister cell formation via ppGpp have not been fully elucidated.

There are several alternative ways to induce persistence. Treatment with antibiotics that reduce transcription, translation, and energy production (ATP) have been shown to increase persistence drastically^5^. In addition, antibiotics like fluoroquinolones that trigger the SOS response via DNA strand breaks, induce persistence in *E. coli*^29^. Furthermore, pre-treatment with both acid (pH 2.5) and hydrogen peroxide (20 mM) increases persistence by four orders of magnitude^30^. Therefore, there are several distinct ways to trigger persister cell formation but the consistent feature is that cells that are less fit have increased persistence^30^.

To explore further mechanisms of persistence, we chose to simplify the system by studying persister cell formation in the absence of ppGpp. Using a selection method that relies on increased persistence, we screened the 4287 gene expression vectors of the ASKA library and identified five novel proteins that increase persistence dramatically. We found that ppGpp is not essential for forming persister cells, and that proteins which simply reduce growth increase persistence in the absence of ppGpp.

## Results

For this work, we primarily utilized ciprofloxacin at 100X the minimum inhibitory concentration (MIC; 0.05 μg/mL)^31^; ciprofloxacin is a fluoroquinolone antibiotic that inhibits DNA replication to kill both growing and non-growing cells, but not persister cells^32^, and is commonly used in persister studies at concentrations well above its MIC^31^,^33^. Previously, we demonstrated^31^ that ciprofloxacin tolerance is due to persistence rather than spontaneous genetic resistance by measuring the tolerance of *E. coli* K-12 cultures after three rounds of ciprofloxacin treatment (5 μg/mL for 3 h) and subsequent regrowth of persisters in fresh media. There was no observable increase in ciprofloxacin survival after each round of regrowth and no colonies were detectable on agar plates with 5 μg/mL ciprofloxacin, showing that there were no resistant cells^31^. Therefore, throughout this work, the number of ciprofloxacin-tolerant cells indicates the number of persister cells.

### ppGpp is not essential for persistence

**Figure 1:**
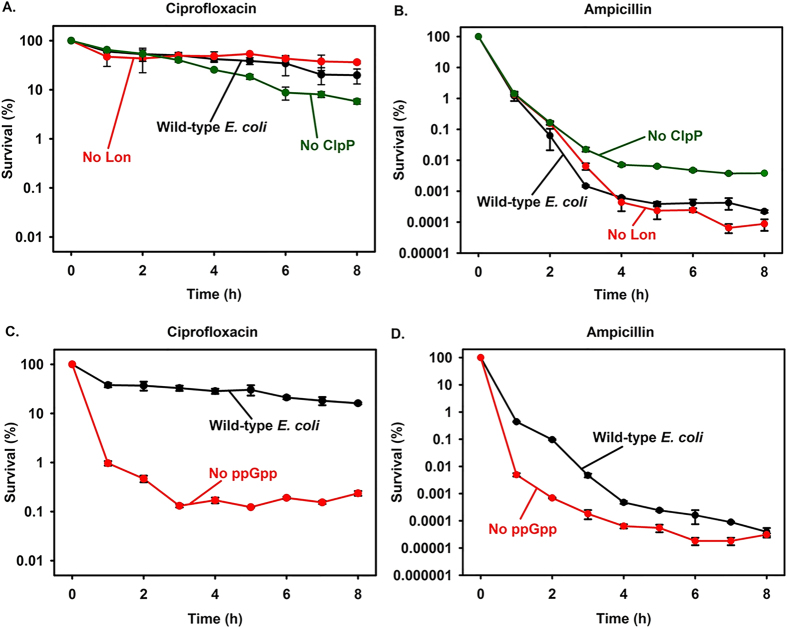
Role of Lon, Clp, and ppGpp in persister cells. Ciprofloxacin (5 μg/mL) and ampicillin (100 μg/mL) were added to stationary-phase cells and exponential-phase cells, respectively, and hourly measurements of cell viability were obtained. (**A**,**B**) Persister cell formation in BW15113 Δ*lon* (“No Lon”) and BW15113 Δ*clpP* (“No ClpP”). Host BW25113 is “Wild-type *E. coli*”. (**C**,**D**) Persister cell formation in the absence of ppGpp using MG1655 (“wild-type *E. coli*”) and the ppGpp-minus strain (MG1655 Δ*relA* Δ*spoT*, “No ppGpp”). Four independent cultures were tested. Means and standard deviations for two representative cultures are shown.

To further explore the mechanisms by which cells become persistent, we initially investigated the role of Lon, Clp, and ppGpp with persistence to ciprofloxacin and ampicillin. The absence of Lon had little impact on the number of persister cells with both antibiotics (Fig. 1A,B), and the absence of Clp reduced tolerance 1.9 ± 0.3 fold with ciprofloxacin (Fig. 1A) and increased tolerance to ampicillin (3 ± 2 to 17 ± 2 fold) (Fig. 1B). Therefore, Lon and Clp protease are not important for creating persister cells under the utilized growth conditions.

Since Lon and Clp proteases were not important for persister cell formation, we tested persistence in the absence of ppGpp by using an *E. coli* Δ*relA* Δ*spoT* mutant previously shown to be devoid of ppGpp^34^. We found that in the absence of ppGpp, tolerance to ciprofloxacin is reduced about 100-fold (Fig. 1C), and tolerance to ampicillin is not affected after long times, 8 h (Fig. 1D). Critically, persister cells still form with both antibiotics. Hence, ppGpp is not essential for forming persister cells.

### Production of toxins increases persistence in the absence of ppGpp

To obtain further evidence that ppGpp is not essential for forming persister cells in *E. coli*, we tested whether the production of various toxins of TA systems still increased persistence in the ppGpp-minus strain. We used ampicillin at 100 μg/mL (10X MIC) for 3 h because ampicillin was used for previous persister studies with toxin expression^8^,^20^,^35^. In addition, we tested ciprofloxacin tolerance at 5 μg/mL (100X MIC), since persister cells have different tolerances to different antibiotics^22^,^36^. For all the strains tested, the activity of each toxin was verified by demonstrating inhibition of cell growth.

**Figure 2:**
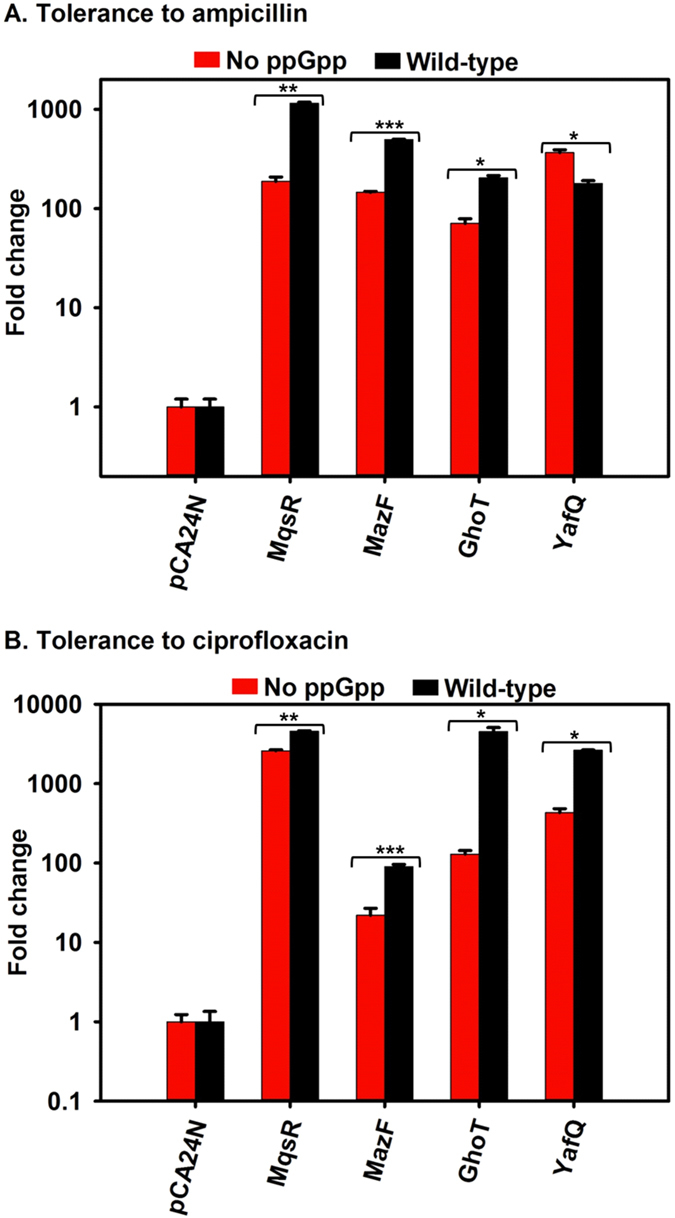
Toxins of TA systems increase persistence in the absence of ppGpp. Ampicillin (100 μg/mL) and ciprofloxacin (5 μg/mL) were added to exponentially-growing cells after 2 h induction of the toxins. “No ppGpp” indicates the MG1655 Δ*relA* Δ*spoT* strain (red bars) and “Wild-type” indicates MG1655 (black bars). Fold changes indicate the increase in persistence relative to the strains harboring the empty plasmid pCA24N. Means and standard deviations for two independent cultures are shown. Statistical analysis was performed by Student’s unpaired two-tailed t-test (*p < 0.05; **p < 0.005; and ***p < 0.0005).

For the ribosome-independent toxin MqsR^37^, which cleaves mRNA at 5′-GCU-3′ sites, the increase in tolerance to ampicillin and ciprofloxacin was higher with ppGpp (1,150 ± 30 fold and 4,586 ± 74, respectively) vs. without ppGpp (188 ± 20 fold and 2,600 ± 80 fold, respectively) (p < 0.005) (Fig. 2A,B). For another ribosome-independent toxin, MazF, which cleaves mRNA at 5′-ACA-3′ sites^38^, the increase in tolerance to ampicillin and ciprofloxacin was also higher with ppGpp (497 ± 2 fold and 2,641 ± 22 fold, respectively) vs. without ppGpp (146 ± 3 fold and 433 ± 50 fold, respectively) (p < 0.0005) (Fig. 2A,B). For the membrane-damaging toxin GhoT^35^,^39^, the same trend of higher tolerance to ampicillin and ciprofloxacin was seen with ppGpp (203 ± 12 fold and 91 ± 6 fold, respectively) than without ppGpp (71 ± 8 fold and 22 ± 5 fold, respectively) (p < 0.05) (Fig. 2A,B). For the endoribonuclease YafQ, which blocks translation elongation through mRNA cleavage at in-frame 5′-AAA-G/A-3′ sequences via its association with the 50S ribosomal subunit^8^,^40^, the increase in tolerance to ampicillin was higher without ppGpp (370 ± 24 fold) than with ppGpp (180 ± 12 fold) (p < 0.05), however, the tolerance to ciprofloxacin was higher with ppGpp (4,545 ± 534 fold) than without ppGpp (130 ± 4) (p < 0.05) (Fig. 2A,B). Critically, for all four of the toxins tested, production of the toxins led to substantial increases in persistence in the absence of ppGpp, which demonstrates that ppGpp is not necessary for persistence with these toxins.

### Proteins that reduce growth increase persistence in the absence of ppGpp

To discern insights into the mechanism by which cells become persistent in the absence of ppGpp, we reasoned that persistence would increase in the Δ*relA* Δ*spoT* strain if a persistence-inducing protein is produced. Hence, we pooled the ASKA library of *E. coli* proteins produced in the backbone pCA24N under control of the *lac* promoter (IPTG-inducible), electroporated them into the Δ*relA* Δ*spoT* strain, and isolated cells that were enriched through multiple rounds of treatment with ciprofloxacin (5 μg/mL); ciprofloxacin kills non-persister cells^41^ so the strains producing the proteins most important for persistence were selected. Specific proteins were identified by plating the persisters and sequencing their ASKA plasmids.

**Figure 3:**
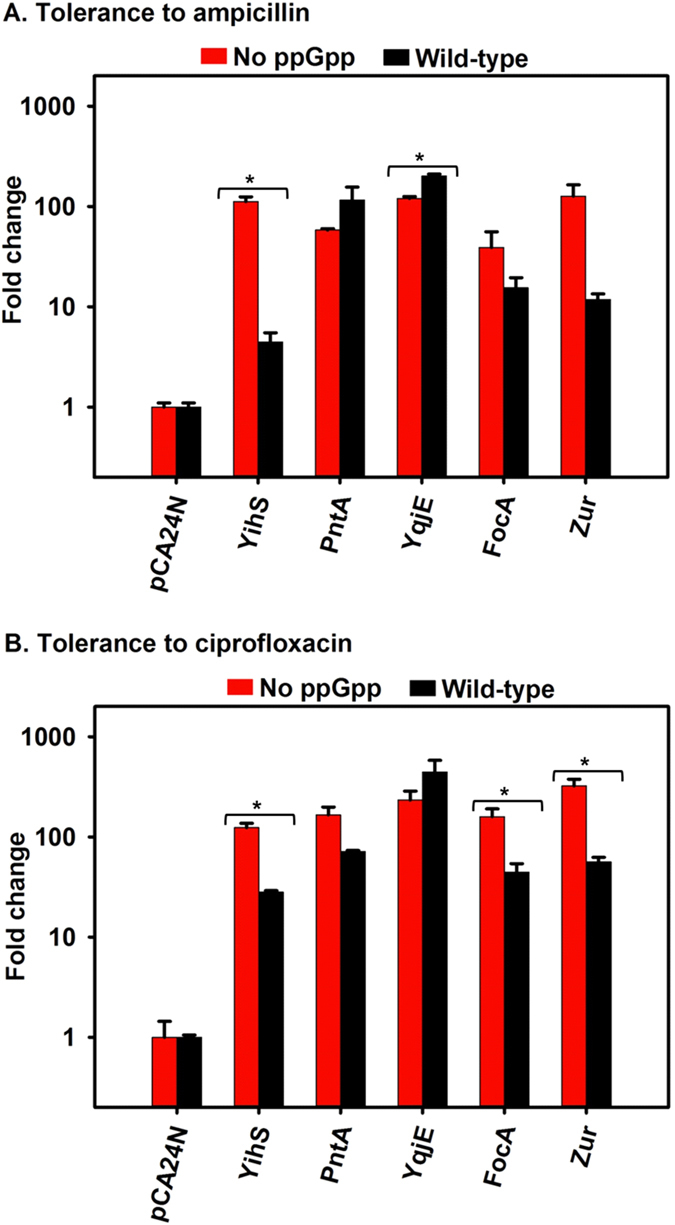
Toxic proteins of non-TA systems increase persistence in the absence of ppGpp. Ampicillin (100 μg/mL) and ciprofloxacin (5 μg/mL) were added to exponentially-growing cells after inducing production of the toxic proteins for 2 h. “No ppGpp” indicates the MG1655 Δ*relA* Δ*spoT* strain (red bars) and “Wild-type” indicates MG1655 (black bars). Fold change indicates the increase in persistence relative to the strains harboring the empty plasmid pCA24N. Means and standard deviations for two independent cultures are shown. Statistical analysis was performed by Student’s unpaired two-tailed t-test (*p < 0.05).

Eight different insert sizes were found out of the 30 colonies analyzed by polymerase chain reaction (PCR) screening; the inserts ranged from about 450 bp to 1700 bp compared to about 200 bp for the empty vector pCA24N. Sequencing identified plasmids containing sequences for *yihS*^+^, *pntA*^+^, *yqjE*^+^, *focA*^+^, *zur*^+^, *mazF*^+^, *yhrC*^+^ (pseudogene), and the intergenic region between *fepB* and *entC. yihS*^+^ (n = 3, 10%), *pntA*^+^ (n = 2, 7%)*, focA*^+^ (n = 2, 7%), and *mazF*^+^ (n = 2, 7%) were nearly evenly distributed, whereas *yqjE*^+^ (n = 6, 20%) and *zur*^+^ (n = 7, 23%) and the intergenic region (n = 7, 23%) were present at higher frequencies. The pseudogene *yrhC* (n = 1, 3%) was present with a lower frequency. For simplicity, pseudogene *yrhC*^+^ and the intergenic region were not used for further study. Therefore, using this selection technique, we identified five new proteins (YihS, PntA, YqjE, FocA, and Zur) important for increasing persistence dramatically in the absence of ppGpp (Fig. 3). We also identified toxin MazF of the MazF/MazE TA system which verified our approach since MazF has been shown to increase persistence^42^, and we demonstrated this persistence in the ppGpp-minus background (Fig. 2).

YihS is an isomerase involved in metabolism of sulfoquinovose, a sulfonic acid derivative of glucose^43^; producing YihS increased tolerance to both ampicillin and ciprofloxacin more without ppGpp (112 ± 13 fold and 124 ± 13 fold, respectively) compared to with ppGpp (4 ± 1 fold and 28 ± 1 fold, respectively), (p < 0.05). Notably, YihS is not toxic in the wild-type strain and is only toxic in the ppGpp-minus strain. PntA is a membrane bound proton-translocating transhydrogenase, which couples reduction of NADP with NADH^44^; the increase in tolerance to ampicillin and ciprofloxacin from PntA was comparable with ppGpp (116 ± 41 fold and 71 ± 2 fold, respectively) and without ppGpp (58 ± 2 fold and 166 ± 33 fold, respectively). YqjE is a putative inner membrane protein; it also increased tolerance similarly with and without ppGpp to both ampicillin (201 ± 9 fold with ppGpp vs. 120 ± 5 fold without ppGpp) and ciprofloxacin (445 ± 136 fold with ppGpp vs. 233 ± 53 fold without ppGpp). Similarly, for FocA, which is a pH-dependent bidirectional formate transport channel^45^, the increase in tolerance to ampicillin was comparable with ppGpp (15 ± 4 fold) and without ppGpp (39 ± 17 fold), and the tolerance to ciprofloxacin was higher without ppGpp (159 ± 31 fold) compared to with ppGpp (44 ± 10 fold) (p < 0.05). Zur is a repressor of zinc transporters ZnuABC^46^ and ZinT^47^. Like FocA, the increase in tolerance to ampicillin by Zur was comparable with ppGpp (12 ± 2 fold) and without ppGpp (127 ± 38 fold) and tolerance to ciprofloxacin was higher without ppGpp (324 ± 53 fold) compared to with ppGpp (56 ± 6 fold) (p < 0.05) (Fig. 3). Therefore, all the identified proteins increased persistence for both antibiotics with or without ppGpp.

**Figure 4:**
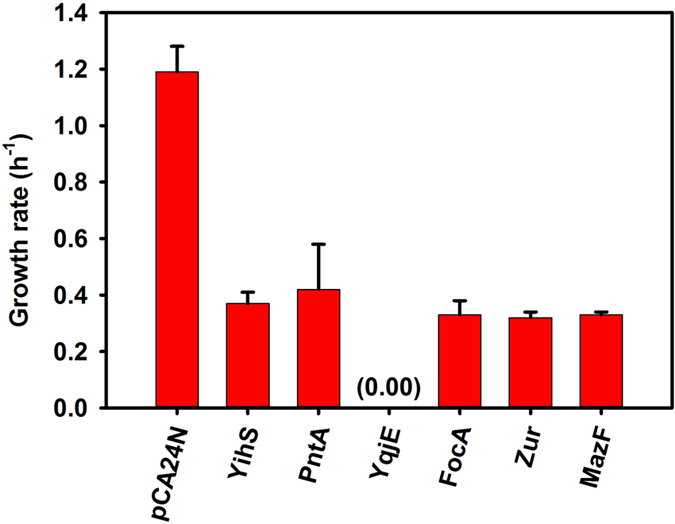
Toxic proteins of non-TA systems reduce the growth rate in the absence of ppGpp. Growth rates of MG1655 Δ*relA* Δ*spoT* carrying the empty vector pCA24N and pCA24N containing genes for newly-identified, non-TA toxic proteins (YihS, PntA, YqjE, FocA, and Zur). MazF, a TA toxin (also found during ASKA screening) was used as a positive control. Means and standard deviations for at least two independent cultures are shown.

To confirm that the increased persistence without ppGpp was due to reduced growth, the growth rates during production of these proteins in the ppGpp-minus strain were determined and found to be significantly lower in the ppGpp-minus strain with these five proteins, compared to the empty vector pCA24N control (Fig. 4, Table S1). The growth rates of the ppGpp-minus, ∆*lon*, and ∆*clpP* strains were also determined, and these strains grew slightly slower compared to the wild-type strain (Fig. S1, Table S1). As expected, the toxin MazF reduced growth (Fig. 4, Table S1). Therefore, the five new persister-inducing proteins identified in the absence of ppGpp by the ASKA selection method reduced growth dramatically.

## Discussion

Our results indicate clearly that persister cells form in the absence of ppGpp. The implication of this finding is that if current drugs focus on reducing ppGpp to reduce persisters, these multi-drug tolerant bacteria will still form, and when they revive, reconstitute an infection. Hence, approaches that go beyond a reduction in ppGpp are needed.

Our results are in stark contrast to the current model which maintains that ppGpp is required for persistence^25^. Our results also refute that Lon protease is required for persistence^48^. Clearly we were able to make persister cells, although at reduced levels, in the absence of ppGpp, and Lon (and Clp) had little impact on persister cell formation in our assays. In contrast, our results with toxins MqsR, MazF, GhoT, and YafQ are consistent with those of the same group which found toxins MazF, RelB, and YafO increase persistence without ppGpp, polyphosphate, and Lon^49^. Clearly the current model which states that persistence occurs through ppGpp, polyphosphate, and Lon holds only for specialized cases (e.g., weak toxins like HipA) and is (i) not suitable for most type 2 TA systems (e.g., toxins MqsR, YafQ, MazF, RelB, and YafO), (ii) not suitable for type 1 TA systems (RNA antitoxin)^28^, (iii) not suitable for type 3 TA systems (RNA antitoxin), and (iv) not suitable for type 5 TA systems (RNase antitoxin) including GhoT. Moreover, a recent report found that the type 2 TA system YefM/YoeB is independent of ppGpp and polyphosphate^27^.

In addition to well-characterized toxins of TA systems that increased persistence in the absence of ppGpp, we identified several proteins from our search of all *E. coli* proteins that increased persistence; these proteins (YihS, PntA, YqjE, FocA, and Zur) have never been linked previously to persistence. The distinguishing feature of these proteins is that all of these proteins increase persistence simply by reducing cell growth. Given that many labs have searched largely in vain for a coherent cause of persistence using such techniques as transposon-sequencing^12^, protein expression^50^, and gene knockouts^51^,^52^, it seems reasonable to conclude that the propensity of a cell to enter the persister state is simply inversely related to its growth rate. This explains why the cell activates production of toxins from TA systems; i.e., as a response to stress, cells reduce growth and a subpopulation becomes persistent. Furthermore, our results fit well with those found previously that show decreasing growth in a chemostat leads to greater persistence; using three antibiotics (ciprofloxacin, tetracycline, and benzyldimethyl-tetradecyl ammonium chloride) the authors^53^ found persistence could be increased up to 90–100% by reducing the specific growth rate to 0.1 h^−1^. Our results are also consistent with the finding that proteins that are unrelated to TA system toxins that are toxic, such as *E. coli* DnaJ of the DnaJ/DnaK/GrpE chaperon system and PmrC of *Salmonella enterica* serovar Typhimurium, also increase persistence in the presence of ppGpp^54^. Therefore, persistence increases as growth decreases, and persistence is not solely dependent on ppGpp.

## Methods

### Bacterial strains and growth media

The bacterial strains and plasmids used are listed in Table 1. *E. coli* strains were routinely grown in LB^55^. Chloramphenicol (Cm; 30 μg/mL) was utilized to maintain the pCA24N-based plasmids^39^ and all strains were grown at 37 °C. The Δ*relA* Δ*spoT* mutation was confirmed via PCR and by DNA sequencing using primers (Rel F: 5′-ACGCTGGCTCGGGATAGC, Rel R: 5′-CATCCACCAGGTCAATCTTCAC, SpoT F: 5′-CGCTGGTACCGGAAGAAAAC, SpoT R: 5′-CACCTGTACCCAGCTGTTACTACC). To ensure the correct genotype, the overnight cultures of Δ*relA* Δ*spoT* cells were always checked for lack of growth on minimal medium plates by washing with normal saline, diluting 1,000 fold (to avoid nutrient especially amino acids carry over), plating on M9 glucose^56^ (0.4%) agar, and incubating for 18 h. The same culture grew after 10^−6^ dilution on LB agar plates, and wild-type MG1655 grew well on the M9 glucose (0.4%) agar plates. This indicates that the Δ*relA* Δ*spoT* is a true mutant having a ppGpp-minus phenotype. To confirm the Δ*relA* Δ*spoT* strains producing toxins were correct, cell growth was checked by plating cells containing plasmid pCA24N and derivatives on LB Cm30 + 1 mM IPTG agar plates as well as on M9 glucose (0.4%) agar plates to show that strains with the toxins did not grow on either of these two plates, confirming the toxin toxicity as well as ppGpp-minus phenotype.

### Persister assay

For strains without plasmids, cells were grown to the late-stationary phase (16 h, 250 rpm) for ciprofloxacin treatments or to the exponential phase (16 h grown culture diluted 1:1000 and regrown to a turbidity of ~0.8 at 600 nm) for ampicillin treatments in 25 mL of LB media. A 1 mL culture was used for a drop assay (10 μL × 3) to check cell viability at 0 h. The remaining stationary phase and exponential phase cultures were treated with ciprofloxacin (5 μg/mL) and ampicillin (100 μg/mL), respectively, shaken at 250 rpm, and the cell viability was tested every hour by taking 1 mL of culture, centrifuging at 10,000 rpm for 3 min, resuspending the cells in 1 mL of normal saline (0.85% NaCl), and performing a drop assay. At least two independent cultures were used. For strains harboring plasmids, cells were diluted to a turbidity of ~0.05 at 600 nm with 25 mL of LB broth and incubated with shaking at 250 rpm until reaching a turbidity of ~0.2. The cultures were then induced with 1 mM IPTG for 2 h at 250 rpm. The cells were harvested by centrifugation at 8,000 rpm for 5 min and resuspended in LB broth to remove IPTG. A 1 mL aliquot was serially diluted for a drop assay to check cell viability. The culture (5 mL) was treated with 100 μg/mL ampicillin and with 5 μg/mL ciprofloxacin for 3 h at 250 rpm. After 3 h, 1 mL of culture was centrifuged at 10,000 rpm for 3 min, the supernatant was discarded, and the cells were resuspended in 1 mL of normal saline. Then, the cell suspension was serially diluted for a drop assay to check cell viability.

### ASKA library selection method

Δ*relA* Δ*spoT* cells were transformed with 40 ng of pooled ASKA plasmid DNA, and cells were allowed to recover by incubating in 1 mL of SOC media for 1 h at 250 rpm. For the first round of selection, 1 mL of culture was inoculated into 25 mL of LB broth and incubated at 250 rpm until reaching a turbidity at 600 nm of ~0.2 followed by induction with 0.2 mM IPTG until reaching a turbidity of ~0.8. The culture was treated with 5 μg/mL of ciprofloxacin for 3 h at 250 rpm and 1 mL was taken for plating. For the second and third rounds of selection, cells from the previous round were washed, diluted 1:100 into 25 mL of fresh LB, and incubated overnight at 250 rpm. Cultures were diluted to a turbidity of ~0.05 at 600 nm in fresh LB and incubated at 250 rpm until reaching a turbidity of ~0.2. Then, as in the first round, cells were induced with IPTG, treated with ciprofloxacin, and 1 mL of culture was used for plating. Thirty colonies were randomly screened by PCR with pCA24N-specific primers to check the insert (Forward: 5′-GCCCTTTCGTCTTCACCTCG-3′ and Reverse: 5′-GAACAAATCCAGATGGAGTTCTGAGGT-3′). pCA24N (empty vector) DNA was used to compare the insert size. Colonies yielding PCR products with different insert sizes (higher than pCA24N) were sequenced and the genes were identified using a BLAST search against the BW25113 genome sequence. At least two colonies with the same insert sizes were sequenced.

### Growth rates

For strains with plasmids, overnight cultures were diluted to a turbidity of ~0.05 at 600 nm and incubated with shaking (250 rpm). IPTG (1 mM) was added at a turbidity of ~0.2 (0 h) to induce the gene on the pCA24N plasmid, and the turbidity was monitored. For strains without a plasmid, overnight cultures were 1:1000 diluted and incubated with shaking (250 rpm) followed by monitoring of turbidity. At least two independent cultures were used.

## Additional Information

**How to cite this article**: Chowdhury, N. *et al*. Persistence Increases in the Absence of the Alarmone Guanosine Tetraphosphate by Reducing Cell Growth. *Sci. Rep.* **6**, 20519; doi: 10.1038/srep20519 (2016).

## Supplementary Material

Supplementary Information

## Figures and Tables

**Table 1:** Bacterial strains and plasmids used in this study.

Strain	Genotype	Source
*E. coli* K-12 BW25113	*rrnB3* Δ*lacZ4787 hsdR514* Δ(*araBAD*)*567* Δ(*rhaBAD*)*568 rph*-*1*	57
BW25113 Δ*lon*	BW25113 Δ*lon* Ω Km^R^	57
BW25113 Δ*clpP*	BW25113 Δ*clpP* Ω Km^R^	57
*E. coli* K-12 MG1655	Wild-type	58
MG1655 Δ*relA* Δ*spoT*	MG1655 Δ*relA* Δ*spoT*	34
Plasmid		
pCA24N	Cm^R^; *lacI*^q^, pCA24N	59
pCA24N-*mqsR*	Cm^R^; *lacI*^q^, pCA24N P_T5-lac_::*mqsR*	59
pCA24N-*mazF*	Cm^R^; *lacI*^q^, pCA24N P_T5-lac_::*mazF*	59
pCA24N-*ghoT*	Cm^R^; *lacI*^q^, pCA24N P_T5-lac_::*ghoT*	59
pCA24N-*yafQ*	Cm^R^; *lacI*^q^, pCA24N P_T5-lac_::*yafQ*	59
pCA24N-*yihS*	Cm^R^; *lacI*^q^, pCA24N P_T5-lac_::*yihS*	59
pCA24N-*pntA*	Cm^R^; *lacI*^q^, pCA24N P_T5-lac_::*pntA*	59
pCA24N-*yqjE*	Cm^R^; *lacI*^q^, pCA24N P_T5-lac_::*yqjE*	59
pCA24N-*focA*	Cm^R^; *lacI*^q^, pCA24N P_T5-lac_::*focA*	59
pCA24N-*zur*	Cm^R^; *lacI*^q^, pCA24N P_T5-lac_::*zur*	59
