# Supplementary material for: Persistence Increases in the Absence of the Alarmone Guanosine Tetraphosphate by Reducing Cell Growth
Source: Sci Rep. 2016 Feb 3;6:20519. doi: 10.1038/srep20519 (PMC4738310; doi:10.1038/srep20519)
Supplement: Supplementary Information [file srep20519-s1.pdf]

SUPPLEMENTARY INFORMATION

Persistence Increases in the Absence of the Alarmone Guanosine Tetraphosphate by Reducing Cell Growth

Nityananda Chowdhury[1], Brian W. Kwan[1], and Thomas K. Wood[1, 2, *]

[1]Department of Chemical Engineering and [2]Department of Biochemistry and Molecular Biology, Pennsylvania State University, University Park, Pennsylvania, 16802-4400, USA

*Correspondence: E-mail twood@engr.psu.edu; Tel.(+)1 814-863-4811; Fax (1) 814-865-7846

Table S1. Specific growth rates. Growth as indicated by turbidity at 600 nm was used to determine the specific growth rate. Bacteria were grown in LB media at 37°C with shaking (250 rpm). The data points used to calculate the specific growth rate are highlighted in orange. Mean and standard deviation (SD) of two independent cultures are shown.

Strain	[a]Turbidity at 600 nm at different time points (h)							Growth rate (h^{-1})	
									Mean ± SD
Δ*relA* Δ*spoT*	0.123 (0 h)	0.184 (0.33 h)	0.266 (0.66 h)	0.414 (1 h)	0.570 (1.33 h)	0.717 (1.66 h)	1.22 (2 h)	**1.13**	**1.19 ± 0.09**
/pCA24N	0.088 (0 h)	0.141 (0.33 h)	0.216 (0.66 h)	0.321 (1 h)	0.496 (1.33 h)	0.642 (1.66 h)	1.01 (2 h)	**1.26**	
Δ*relA* Δ*spoT*	0.136 (0 h)	0.366 (1 h)	0.670 (2 h)	0.800 (3 h)	1.21 (4 h)	2.12 (5 h)		**0.40**	**0.37 ± 0.04**
/pCA24N-*yihS*	0.130 (0 h)	0.360 (1 h)	0.540 (2 h)	0.690 (3 h)	1.01 (4 h)	1.92 (5 h)		**0.34**	
Δ*relA* Δ*spoT*	0.146 (0 h)	0.245 (1 h)	0.240 (2 h)	0.320 (3 h)	0.610 (4 h)	1.18 (5 h)		**0.30**	**0.42 ± 0.16**
/pCA24N-*pntA*	0.170 (0 h)	0.303 (1 h)	0.620 (2 h)	0.880 (3 h)	1.35 (4 h)	1.94 (5 h)		**0.53**	
[b]Δ*relA* Δ*spoT*	0.160 (0 h)	0.177 (1 h)	0.120 (2 h)	0.080 (3 h)	0.050 (4 h)	0.040 (5 h)	0.040 (6 h)	**0.00**	**0.00 ± 0.00**
/pCA24N-*yqjE*	0.143 (0 h)	0.145 (1 h)	0.100 (2 h)	0.090 (3 h)	0.080 (4 h)	0.110 (5 h)	0.140 (6 h)	**0.00**	
Δ*relA* Δ*spoT*	0.134 (0 h)	0.225 (1 h)	0.270 (2 h)	0.370 (3 h)	0.480 (4 h)	0.810 (5 h)	1.28 (6 h)	**0.37**	**0.33 ± 0.05**
/pCA24N-*focA*	0.116 (0 h)	0.210 (1 h)	0.520 (2 h)	0.670 (3 h)	0.940 (4 h)	1.63 (5 h)	2.05 (6 h)	**0.30**	
Δ*relA* Δ*spoT*	0.120 (0 h)	0.280 (1 h)	0.443 (2 h)	0.565 (3 h)	0.778 (4 h)	1.45 (5 h)	2.12 (6 h)	**0.34**	**0.32 ± 0.02**
/pCA24N-*zur*	0.116 (0 h)	0.257 (1 h)	0.376 (2 h)	0.491 (3 h)	0.646 (4 h)	1.05 (5 h)	1.65 (6 h)	**0.31**	
Δ*relA* Δ*spoT*	0.168 (0 h)	0.227 (1 h)	0.199 (2 h)	0.199 (3 h)	0.247 (4 h)	0.398 (5 h)	0.782 (6 h)	**0.34**	**0.33 ± 0.01**
/pCA24N-*mazF*	0.120 (0 h)	0.131 (1 h)	0.117 (2 h)	0.194 (3 h)	0.344 (4 h)	0.502 (5 h)	0.669 (6 h)	**0.33**	
MG1655	0.005 (0 h)	0.073 (2 h)	0.118 (2.33 h)	0.209 (2.66 h)	0.356 (3 h)	0.513 (3.33 h)	0.786 (3.66 h)	**1.65**	**1.75 ± 0.15**
	0.005 (0 h)	0.066 (2 h)	0.119 (2.33 h)	0.225 (2.66 h)	0.376 (3 h)	0.537 (3.33 h)	0.787 (3.66 h)	**1.86**	
Δ*relA* Δ*spoT*	0.004 (0 h)	0.047 (2.5 h)	0.088 (2.83 h)	0.140 (3.17 h)	0.223 (3.5 h)	0.378 (3.83 h)	0.463 (4.17 h)	**1.39**	**1.36 ± 0.04**
	0.004 (0 h)	0.060 (2.5 h)	0.107 (2.83 h)	0.170 (3.17 h)	0.253 (3.5 h)	0.406 (3.83 h)	0.518 (4.17 h)	**1.33**	
BW25113	0.005 (0 h)	0.091 (2 h)	0.171 (2.33 h)	0.325 (2.66 h)	0.508 (3 h)	0.791 (3.33 h)		**1.93**	**1.92 ± 0.02**
	0.006 (0 h)	0.101 (2 h)	0.184 (2.33 h)	0.355 (2.66 h)	0.523 (3 h)	0.815 (3.33 h)		**1.90**	
Δ*lon*	0.005 (0 h)	0.077 (2 h)	0.130 (2.33 h)	0.238 (2.66 h)	0.523 (3 h)	0.599 (3.33 h)	0.814 (3.66 h)	**1.71**	**1.65 ± 0.08**
	0.005 (0 h)	0.064 (2 h)	0.105 (2.33 h)	0.183 (2.66 h)	0.404 (3 h)	0.471 (3.33 h)	0.692 (3.66 h)	**1.59**	
Δ*clpP*	0.005 (0 h)	0.073 (2.5 h)	0.131 (2.83 h)	0.212 (3.17 h)	0.351 (3.5 h)	0.538 (3.83 h)	0.777 (4.17 h)	**1.47**	**1.50 ± 0.04**
	0.005 (0 h)	0.064 (2.5 h)	0.116 (2.83 h)	0.191 (3.17 h)	0.324 (3.5 h)	0.497 (3.83 h)	0.701 (4.17 h)	**1.53**	

[a]For strains with the empty vector or vector with toxic protein genes, 0 h time point is the time when 1 mM IPTG was added (OD_{600} ~0.2).
[b]Growth rate of this strain was 0 (zero) as no increase in turbidity was observed.

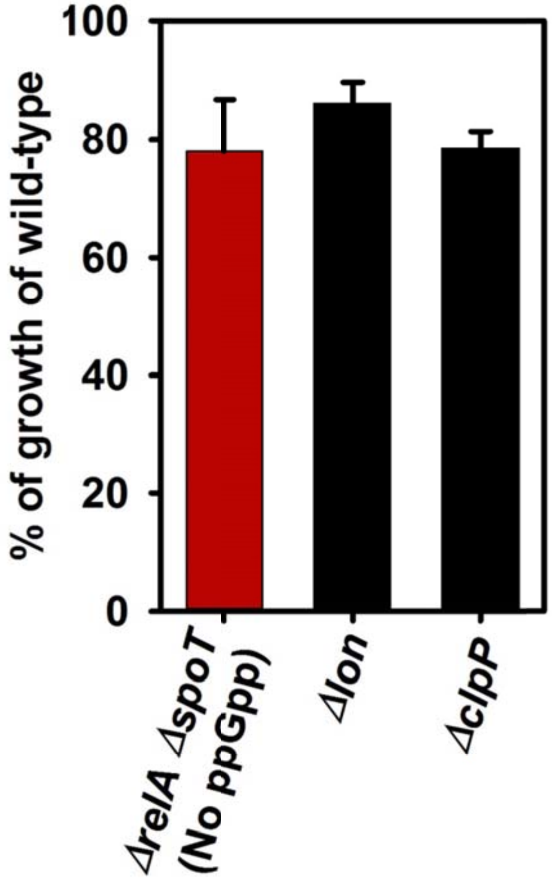

Figure S1. Relative specific growth rates of the Δ*relA* Δ*spoT* (No ppGpp), Δ*lon*, and Δ*clpP* strains. The percentage of growth of each strain was calculated by normalizing by the specific growth rate of the respective parent strains (**Table S1**), e.g., MG1655 for the Δ*relA* Δ*spoT* strain (red bar) and BW25113 for Δ*lon* and Δ*clpP* (black bars). Overnight (16 h) grown cultures were diluted 1:1000 in fresh LB media, incubated at 37°C and 250 rpm, and the turbidity at 600 nm was monitored to obtain the specific growth rate. Means and standard deviations for two independent cultures are shown.
